# Individual recovery of health-related quality of life during 18 months post-burn using a retrospective pre-burn measurement: an exploratory study

**DOI:** 10.1007/s11136-020-02678-0

**Published:** 2020-10-22

**Authors:** Elise Boersma-van Dam, Rens van de Schoot, Helma W. C. Hofland, Iris M. Engelhard, Nancy E. E. Van Loey

**Affiliations:** 1 Association of Dutch Burn Centres, P.O. Box 1015, 1940 EA Beverwijk, The Netherlands; 2 Department of Clinical Psychology, Utrecht University, Utrecht, The Netherlands; 3 Department of Methodology and Statistics, Faculty of Social and Behavioral Sciences, Utrecht University, Utrecht, The Netherlands; 4 Optentia Research Program, Faculty of Humanities, North-West University, Vanderbijlpark, South Africa; 5 Burn Center Maasstad Hospital, Rotterdam, The Netherlands

**Keywords:** Burns, Quality of life, Post-traumatic stress, Individual recovery, Pre-injury data

## Abstract

**Purpose:**

This study explored the individual trajectories of health-related quality of life (HRQL) compared to recalled pre-burn level of HRQL and investigated whether burn severity and post-traumatic stress disorder (PTSD) symptoms increase the risk of not returning to pre-burn level of HRQL.

**Methods:**

Data were obtained from 309 adult patients with burns in a multicenter study. Patients completed the EQ-5D-3L questionnaire with a Cognition bolt-on shortly after hospital admission, which included a recalled pre-injury measure, and, again, at 3, 6, 12 and 18 months post-burn. Burn severity was indicated by the number of surgeries, and PTSD symptoms were assessed with the IES-R at three months post-burn. Pre- and post-injury HRQL were compared to norm populations.

**Results:**

Recalled pre-injury HRQL was higher than population norms and HRQL at 18 months post-burn was comparable to population norms. Compared to the pre-injury level of functioning, four HRQL patterns of change over time were established: Stable, Recovery, Deterioration, and Growth. In each HRQL domain, a subset of patients did not return to their recalled pre-injury levels, especially with regard to Pain, Anxiety/Depression, and Cognition. Patients with more severe burns or PTSD symptoms were less likely to return to pre-injury level of functioning within 18 months post-burn.

**Conclusion:**

This study identified four patterns of individual change. Patients with more severe injuries and PTSD symptoms were more at risk of *not* returning to their recalled pre-injury HRQL. This study supports the face validity of using a recalled pre-burn HRQL score as a reference point to monitor HRQL after burns.

**Electronic supplementary material:**

The online version of this article (10.1007/s11136-020-02678-0) contains supplementary material, which is available to authorized users.

## Background

Life after burn injury may encompass a range of difficulties, including physical symptoms such as pain and itch, psychological symptoms such as traumatic stress and anxiety and social difficulties such as stigmatization, all of which may affect health-related quality of life (HRQL) for years [1]. HRQL is a widely used concept that encompasses a patient’s perception of one’s health condition on physical, psychological and social functioning [2]. Prior studies in burn populations have usually compared patients’ HRQL with the general population [3, 4] to establish burn-related sequelae. However, improved technological possibilities, such as real-time processing of digitally completed patient-reported outcome measures, have created the means to systematically monitor a patient’s therapeutic progress and make it possible to customize clinical approaches to specific needs [5, 6]. For the purpose of monitoring recovery from the patient’s viewpoint, comparing patients’ level of functioning with their pre-burn level is recommended [7]. Because prospectively collected information on pre-burn HRQL is usually not available in trauma populations, retrospective data collection is indicated [8].

The extant literature on HRQL in burn patients has shown that, on average and compared to norm groups, most HRQL domains are affected shortly after a burn injury and recover over time, except domains such as anxiety, depression and pain [9]. The studies that have investigated recalled pre-burn HRQL, all using the SF-36 questionnaire, showed that, in general, HRQL decreased after the burn injury followed by a gradual increase over time [10–15]. Two small studies investigating HRQL after wildfire, found reduced HRQL at 12 and 36 months compared to recalled pre-injury HRQL [16, 17]. Of notice, the EQ-5D questionnaire has not been used to measure recalled pre-burn HRQL, few studies have included measures beyond 12 months post-burn [11], and individual recovery trajectories have not been described.

A number of predictors of HRQL after burn injuries have been established. Specifically, burn severity, as measured by length of hospital stay and number of surgeries, and psychological factors, such as post-traumatic stress disorder (PTSD) symptoms, are consistently associated with HRQL over time [10, 18, 19]. PTSD is one of the most prevalent mental health problems after a burn injury: around 9% of patients are typically diagnosed with PTSD, about 15% show sub-threshold symptom levels 1 year post-injury, and up to 43% report substantial symptoms 1 year post-burn [20, 21]. The few studies that did assess recalled pre-burn HRQL have shown that, on average, more severely burned patients approach mean pre-burn HRQL level later or stayed at lower HRQL level than less severely burned patients [10, 11, 13, 15]. As these studies focused on group level changes, it is not clear to what extent burn severity and PTSD symptoms are associated with individual trajectories, and specifically whether individuals return to their own (recalled) pre-burn level instead of returning to the group average or population norms.

The current longitudinal study had three aims: (1) to compare recalled pre-burn HRQL (assessed during hospitalization) and post-burn HRQL to population norms; (2) to explore individual patterns of change in HRQL domains assessed over a period of 18 months post-burn relative to recalled pre-burn level; and (3) to examine whether more severe burns and PTSD symptoms were associated with a higher risk of *not* returning to pre-burn HRQL at individual level.

## Methods

### Participants

The data (*N* = 480) from this study came from two larger projects: one focused on pain in three Dutch and two Belgian burn centers (Study 1 [22, 23]) and one focused on the social impact in three Dutch and three Belgian burn centers (Study 2). Patients were recruited from April 2010 to December 2012 in Study 1 and from October 2013 to October 2015 in Study 2. Both cohorts were prospectively followed up for 18 months. Inclusion criteria for patients in both studies were: a hospital admission of > 24 h following the burn event, aged 18 years or older and sufficient command of Dutch. Exclusion criteria were: psychiatric problems that interfere with questionnaire comprehension (e.g., psychosis, cognitive problems), and inhalation injury without external burns.

### Procedure

Patients were invited to participate in the studies by a local researcher during their stay in the burn center. After they received oral and written information about the study, they provided written informed consent. Patients completed T1 and the recalled pre-injury measure during hospitalization following the burn injury. They completed the follow-up assessments by mail at 3 (T2), 6 (T3), 12 (T4) and 18 (T5) months post-burn. The study was approved by institutional review boards in the Netherlands and Belgium (Study 1: NL27996.094.09, B670201112923; Study 2: NL44682.094.13, B670201420373).

### Measures

#### Health-related quality of life

The EQ-5D-3L + Cognition is a self-report scale used to assess generic HRQL. It was completed during hospitalization, including the recalled pre-burn measure, and at 3, 6, 12, and 18 months after the burn injury. HRQL is assessed along six single-item health domains: Mobility, Self-care, Usual Activities, Pain, Anxiety/Depression and Cognition. The added Cognition domain measures to what extent the patient experiences problems with memory and concentration. For each domain, patients reported their health ‘in the past week’ or ‘before the burn event’ (for the recalled pre-burn measure). Answers were rated on a 3-point scale: ‘no problems’, ‘moderate problems’, or ‘severe problems’. The first five domains were combined into the EQ-5D Summary Index based on a scoring algorithm. The Summary Index ranges from − 0.594 ‘worse than death’ through 0 ‘death’ to 1 ‘full health’ [24]. In addition, the EQ-5D includes a Visual Analog Scale (VAS) that is scaled vertically and runs between 0 (worst imaginable health state) and 100 (best imaginable health state). The EQ-5D is short and easy to complete and it has good feasibility and reasonable criterion validity in the burn population [25]. The addition of a Cognition domain slightly improved the psychometric performance of the EQ-5D in traumatic brain injury patients [26].

#### Post-traumatic stress disorder (PTSD) symptoms

PTSD symptoms during hospitalization, at 3 and 6 months post-burn were measured using the validated Dutch version of the Impact of Event Scale-Revised (IES-R; [27, 28]). The IES-R is a self-report questionnaire that measures PTSD symptoms in the past week. The two studies in this research used different scoring systems of the Dutch IES-R. Study 1 used the original scoring of the 15-item version [29] with four answer categories 0 ‘never’, 1 ‘rarely’ 3 ‘sometimes’ and 5 ‘often’. Answers on these 15 items were summed, with scores of 26 and higher indicating a possible diagnosis of PTSD based on symptoms, without taking into account the criterion of functional impairment or suffering due to symptoms. Study 2 used the 22-item scoring system that included also the hyperarousal subscale. Answers were given on a 5-point Likert scale and summed to obtain a total score ranging from 0 to 88, with scores of 33 and higher indicating a possible diagnosis of PTSD. The IES-R has high sensitivity as a screening tool for PTSD after burn injuries [30].

#### Demographic data and injury severity

Age, gender, number of surgeries and total body surface area (TBSA) burned were recorded from the medical file. TBSA is the estimated percentage of the body covered with partial and full thickness burns. Number of surgeries was used as an indicator of burn severity. It indicates the number of skin graft procedures that was required to cover the wounds.

### Statistical analysis

First, pre-burn and post-burn Summary and VAS mean scores in our sample were compared to population norms using *t*-tests. The normative data came from a national representative sample of the non-institutionalized adult population [31]. Effect sizes (Cohen’s *d*) were calculated to quantify the differences between the sample and the population norms. The vast majority of the final sample came from the Netherlands (91.3%; the remainder came from Belgian burn centers), therefore the sample means were compared to Dutch population norms.

Second, for each of the six EQ-5D domains, the Summary Index and the VAS, patients were allocated to a pattern of change in HRQL relative to their recalled pre-burn HRQL. For the six domains, the post-burn item scores at all assessments were directly compared to the recalled pre-burn score to identify a decrease, increase or no change. For the EQ-Summary Index and EQ-VAS, the Minimally Important Difference (MID) was used as an indication of the minimum change that reflects a clinically relevant improvement or deterioration in HRQL. For the EQ-Summary Index, ‘pre-burn level’ was defined by a score as close as 0.074 to pre-burn state, based on the MID in patients with a wide range of medical conditions [32]. For the EQ-VAS, ‘pre-burn level’ was defined by a score as close as 8 to pre-burn state, based on the MID in several studies in specific (non-burn) patient populations [33–35]. The MIDs were established using both anchor and distribution-based methods. Several patients were excluded in the concerning domain analyses because of a floor effect, as their health state could not be (measurably) negatively impacted after the burns. They had severe pre-burn problems in one or more domains (*n*_Mobility_ = 2 *n*_Self-care_ = 3, *n*_Usual Activities_ = 7 *n*_Pain/Discomfort_ = 9, *n*_Anxiety/Depression_ = 2, *n*_Cognition_ = 1), a pre-burn Summary Index ≤ 0 (*n* = 5) or a pre-burn VAS ≤ 10 (*n* = 1). Four patterns were defined: (1) Stable, including patients who did not show any post-burn decline in HRQL (beyond the MID) and who were at their pre-burn level of functioning at 18 months post-burn. (2) Growth, including patients who did not show any post-burn decline in HRQL (beyond the MID) and who showed increased level of HRQL at 18 months post-burn relative to the pre-burn level; (3) Recovery, characterized by a post-burn decline in HRQL followed by recovery to pre-burn level or beyond at 18 months; and (4) Deterioration, characterized by a post-burn decline in HRQL and below pre-burn level functioning at 18 months. If HRQL at 18 months was unknown (*n* = 29–31; 9.4–10.0% of the sample), HRQL at 12 months was used as final outcome.

Third, to study who recovered to pre-burn HRQL and who did not, we selected the individuals attributed to the two patterns Recovery and Deterioration because they showed a decrease in HRQL after the injury, which suggests an effect of the burn injury. Logistic regression analyses were used to study whether burn severity and PTSD symptoms assessed at 3 months post-burn increased the risk to be assigned to the Deterioration pattern. The 3 months assessment was chosen, because symptoms should persist for at least 1 month to be diagnosed as PTSD and three months was the earliest available measurement after that point [36]. For the Summary Index and VAS, *t*-tests were conducted to test whether the mean pre-burn HRQL in the Recovery and Deterioration groups differed. Analyses were performed using IBM SPSS 24. Sample sizes may vary between analyses because of missing data in one of the health domains or PTSD symptoms.

## Results

### Sample and attrition

A total of 480 patients completed the first assessment (T1) and 258 (54%) completed EQ-5D assessments at all follow-up measurements (T2–T5). A total of 169 patients were excluded from the statistical analyses due to missing recalled pre-burn EQ-5D measurements (*n* = 24) or missing EQ-5D measurements at both T4 and T5 (*n* = 145). The excluded patients did not differ from the final sample in terms of gender, *χ*^2^(1) = 0.40, *p* = .54, or PTSD symptoms, *χ*^2^(1) = 2.17, *p* = .16, but were significantly younger, *M* = 38.3 versus 44.8, *t*(478) = 4.44, *p* < .001, Cohen’s *d* = 0.43, and had fewer surgeries, *χ*^2^(2) = 9.81, *p* = .007.

The final sample consisted of 311 patients. They had a mean age of 44.8 years (SD = 15.5), and most were male (*n* = 214, 68.8%). Mean total body surface area (TBSA) burned was 9.7% (SD = 10.0, range 0.40–75.0%). Median number of surgeries was 1 (range 0–14). For further analyses, this variable was recoded into ‘no surgeries’ (*n* = 132; 42.4%), ‘one surgery’ (*n* = 124; 39.9%) or ‘more than one surgery’ (*n* = 55; 17.7%). The number of patients scoring above the IES-R cut-off for a possible PTSD diagnosis at 3 months post-burn was 53 (18.0%). Seventeen patients did not complete the IES-R at 3 months.

### HRQL over time and comparison to population norms

Table 1 shows that the mean recalled pre-burn Summary Index and VAS of the sample were somewhat higher than the general population norms [31]. During hospitalization, the Summary Index dropped to a mean of 0.44 (a reduction of 52.2% compared to pre-burn) and a VAS of 63.7 (a reduction of 25.7%). Over time, on average, HRQL recovered, and 18 months post-burn, the sample means for the Summary Index and VAS were comparable to those in the general population, but still somewhat lower than both the pre-burn Summary Index and VAS (Table 1).

**Table 1:** Descriptives and comparison of pre- and post-burn HRQL and general population norms

Descriptives	Summary Index				VAS			
	*N*	*M*	SD		*N*	*M*	SD	
Population norm		.89				82.0		
Pre-burn	311	.92	.21		301	85.7	13.1	
In hospital	305	.44	.37		300	63.7	21.3	
18 months*	311	.87	.21		301	82.5	15.3	

Comparisons	*t*	*df*	*p*	*d*	*t*	*df*	*p*	*d*
Population norm vs pre-burn	2.63	310	.009	0.14	4.87	300	< .001	0.28
Population norm vs 18 months*	− 1.63	310	.11	0.10	0.59	300	.55	0.03
Pre-burn vs 18 months*	3.63	310	< .001	0.24	3.26	300	.001	0.22

*HRQL* health-related quality of life

*If individuals’ 18 months HRQL was missing, 12 months HRQL was used

### Patterns of change

**Fig. 1:**
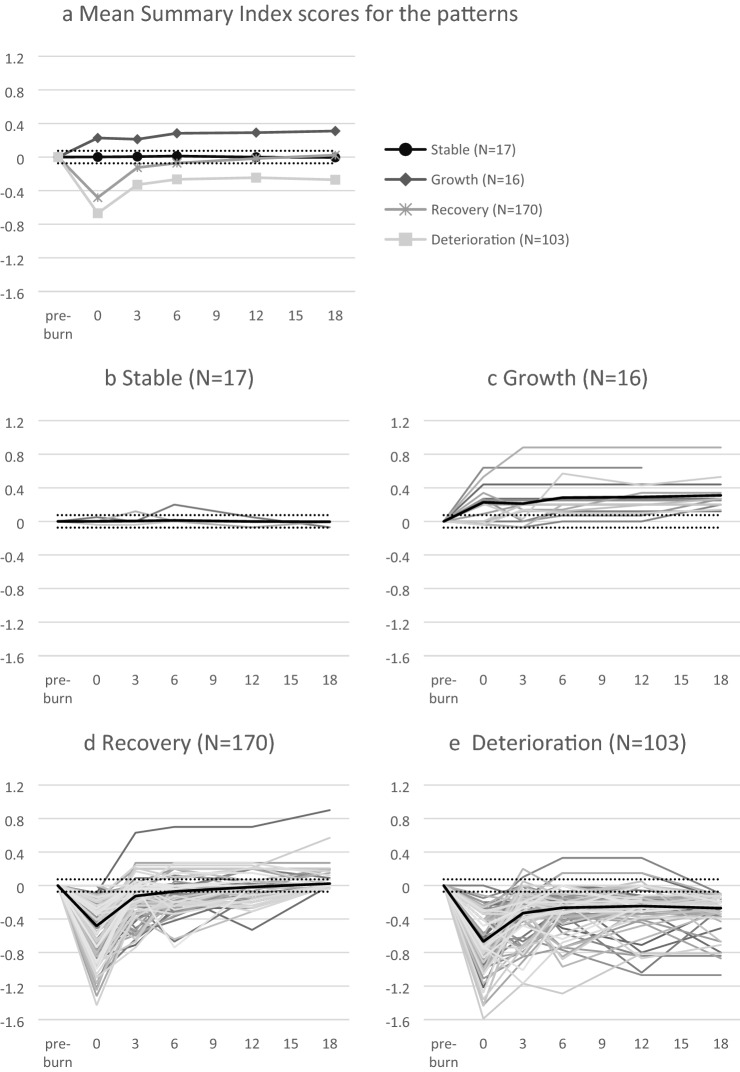
Patterns of health-related quality of life change relative to pre-injury level of functioning during eighteen months post-burn for the EQ-5D Summary Index. Pattern means (panel **a**) and individual trajectories over time per pattern (panel **b**–**e**) are displayed. The black lines (panel **b**–**e**) represent the pattern means; dotted lines represent the MID boundaries

Four patterns of change in HRQL were observed in each domain and on the Summary Index and VAS: Stable, Growth, Recovery and Deterioration. Figure 1a shows the shape and frequency of each pattern over time on the Summary Index relative to pre-burn functioning. Figure 1b–e shows that individual trajectories in the four patterns varied widely. Table 2 presents the frequencies of the four patterns for each EQ-domain and for the Summary Index and VAS separately. For the Summary Index and VAS, the Recovery pattern was most common (55.6% and 48.5% respectively), followed by the Deterioration pattern (33.7% and 35.5% respectively). The Stable and Growth patterns were less common. In the physical domains *Mobility and Self-Care*, the Stable pattern and the Recovery pattern were most frequent, indicating that a substantial number of patients did not show problems in these areas after the injury, and for *Self-Care* only a few patients reported persisting problems (Deterioration). In the physical domains *Usual Activities and Pain/Discomfort*, the percentages of Stable patients were the lowest, indicating that most patients experienced (temporal) problems in these areas. The *Pain/Discomfort* domain was the most troublesome, as it included relatively many patients in the Deterioration pattern. In the *Anxiety/Depression and the Cognition domain,* most patients followed a Stable unaffected pattern. In each domain a subsample of patients showed persistent problems at 18 months post-burn (Deterioration pattern), especially regarding *Pain/Discomfort, Anxiety/Depression, Cognition and Usual Activities*.

**Table 2:** Frequencies of HRQL patterns of change in each domain of the EQ-5D

EQ-5D domain	Stable		Growth		Recovery		Deterioration		Total
	*N*	%	*N*	%	*N*	%	*N*	%	*N*
Summary	17	5.6	16	5.2	170	55.6	103	33.7	306
VAS	22	7.4	26	8.7	145	48.5	106	35.5	299
Mobility	139	45.0	9	2.9	136	44.0	25	8.1	309
Self-care	107	34.7	6	1.9	187	60.7	8	2.6	308
Usual activities	42	13.8	7	2.3	214	70.4	41	13.5	304
Pain/discomfort	44	14.6	19	6.3	165	54.6	74	24.5	302
Anxiety/depression	175	56.6	5	1.6	84	27.2	45	14.6	309
Cognition	163	52.6	10	3.2	89	28.7	48	15.5	310

*HRQL* health-related quality of life

### Recovery status at 18 months in the Recovery and Deterioration pattern

Table 3 shows HRQL outcomes at 18 months compared to pre-injury level for all patients that showed a decrease in HRQL after the injury (i.e., the Recovery or Deterioration patterns). Regarding the Summary Index and VAS, the majority of patients returned to pre-injury level or higher level, but 37.7 and 42.2%, respectively, did not return to pre-burn level. Compared to the domains and Summary Index, the VAS showed the highest number of patients reporting growth beyond pre-injury level (16.7%). Of the individual domains, Self-Care showed the highest recovery rates, whereas in the other domains, 15.5% or more of the patients did not return to pre-injury level within 18 months.

**Table 3:** Number of patients in a Deterioration or Recovery pattern with a health-related quality of life score at 18 months that is below, at, or above pre-burn level

EQ-5D domain	Deterioration		Recovery				Total
	Below pre-burn level		At pre-burn level		Above pre-burn level		
	*n*	%	*n*	%	*n*	%	*n*
Summary	103	37.7	156	57.1	14	5.1	273
VAS	106	42.2	103	41.0	42	16.7	251
Mobility	25	15.5	134	83.2	2	1.2	161
Self-care	8	4.1	186	95.4	1	0.5	195
Daily activities	41	16.1	211	82.7	3	1.2	255
Pain/discomfort	74	31.0	162	67.8	3	1.3	239
Anxiety/depression	45	34.9	83	64.3	1	0.8	129
Cognition	48	35.0	88	64.2	1	0.7	137

### Burn severity, PTSD symptoms and Recovery versus Deterioration

**Fig. 2:**
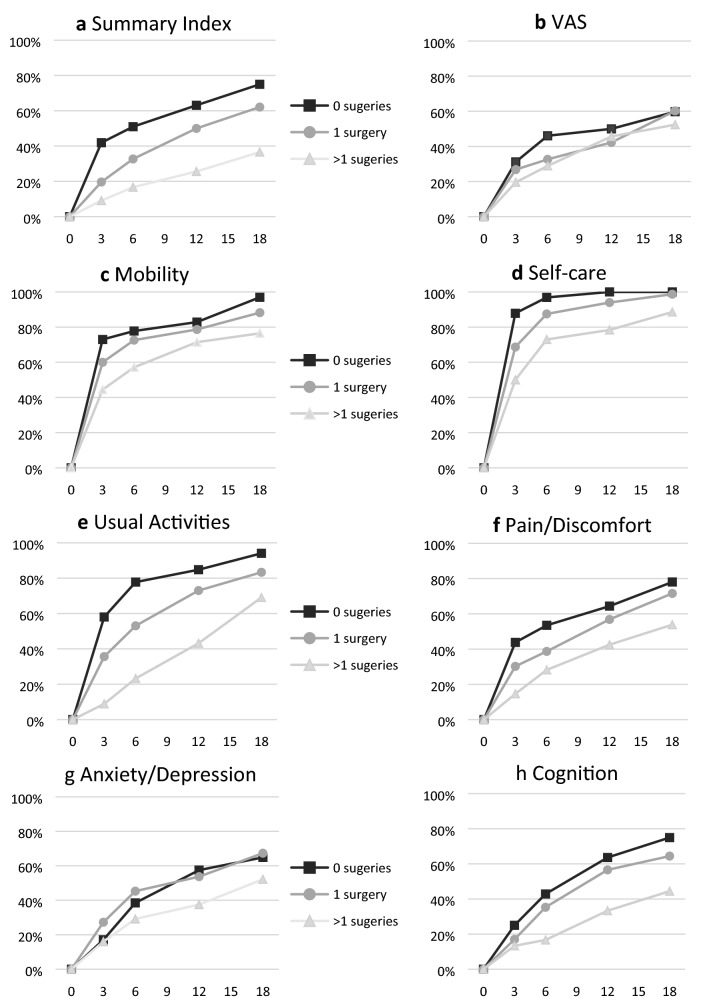
Percentages of patients with three levels of burn severity returning to pre-injury health-related quality of life over time (months) on the Summary Index (panel **a**), VAS (panel **b**), and the six domains (panel **c**–**h**)

**Fig. 3:**
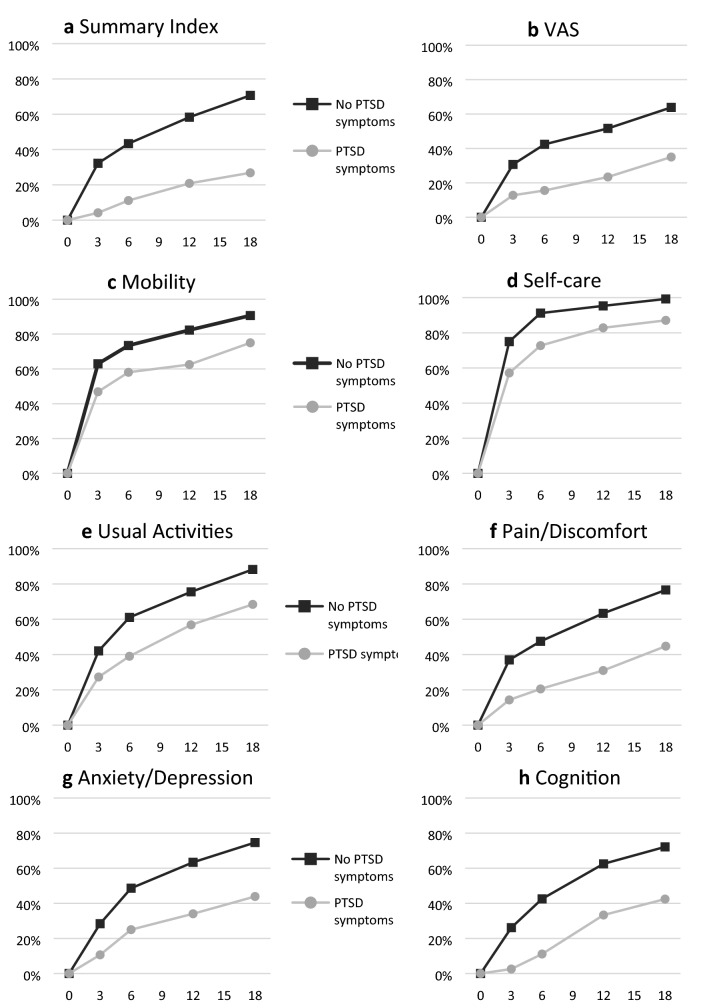
Percentages of patients with and without substantial PTSD symptoms returning to pre-injury health-related quality of life over time (months) on the Summary Index (panel **a**), VAS (panel **b**), and the six domains (panel **c**–**h**)

Figure 2 depicts the percentage of patients that returned to pre-burn HRQL as a function of number of surgeries and Fig. 3 as a function of presence of PTSD symptoms for the Summary Index, VAS, and the individual domains. In most domains and the Summary Index, recovery percentages were highest in the group without surgeries at each time point and lowest in the group with multiple surgeries. Differences between surgery groups seemed small on the VAS. The largest differences in the individual domains between the three groups were observed for Usual Activities, Pain/Discomfort and Cognition. For Anxiety/Depression, the groups without surgery and with one surgery showed similar recovery percentages over time.

Regarding PTSD symptoms, in each domain, Summary Index and VAS, recovery percentages were lower in the group with substantial PTSD symptoms. The largest differences in the individual domains between the two groups were found for Pain/Discomfort, Anxiety/Depression and Cognition.

With logistic regression analyses, the probability of belonging to the Recovery group was regressed on burn severity and PTSD symptoms (see Table 4). For Self-care, the logistic regression was not useful, because of the high recovery rates (see Table 3). For the Summary Index and Usual Activities, the results showed that compared to patients without surgery, patients who needed multiple surgeries were significantly less likely to have recovered to pre-injury level at 18 months (Odds ratio of 3.70 and 3.85 respectively). For the Summary Index, VAS and the respective health domains, patients with substantial PTSD symptoms at 3 months post-burn were less likely to recover to pre-injury level at 18 months than patients without substantial PTSD symptoms (Odds ratios ranged between 2.70 and 5.56). The results for PTSD symptoms shortly after hospital admission and at 6 months post-burn were also explored. Associations were smaller at admission (Odds ratios ranged between 1.56 and 3.23, see Table 5 in the supplementary material) and stronger at 6 months post-burn (Odds ratios ranged between 3.23 and 6.67, see Table 6 in the supplementary material).

**Table 4:** Summary of logistic regression analyses with Recovery to pre-burn level of HRQL in each EQ-5D domain as dependent variables and number of surgeries and PTSD symptoms as independent variables

	*B*	SE	Wald	*df*	*p*	OR	95% CI OR	1/OR
Summary Index *χ*^2^(3) = 38.72, *p* < .001, Nagelkerke *R*^2^ = 0.19								
Surgery			10.92	2	.004			
1 surgery	− 0.35	0.31	1.22	1	.27	0.71	[0.38;1.31]	1.41
> 1 surgeries	− 1.30	0.39	10.83	1	.001	0.27	[0.13;0.59]	3.70
PTSD symptoms	− 1.74	0.36	23.30	1	< .001	0.18	[0.09;0.36]	5.56
VAS *χ*^2^(3) = 10.12, *p* = .02, Nagelkerke *R*^2^ = 0.06								
Surgery			0.54	2	.76			
1 surgery	0.18	0.30	0.35	1	.55	1.19	[0.66;2.16]	0.84
> 1 surgeries	− 0.06	0.37	0.02	1	.88	0.95	[0.46;1.95]	1.05
PTSD symptoms	− 1.03	0.34	9.30	1	.002	0.36	[0.18;0.69]	2.78
Mobility *χ*^2^(3) = 9.29, *p* = .03, Nagelkerke *R*^2^ = 0.11								
Surgery			1.74	2	.42			
1 surgery	− 0.05	0.65	0.01	1	.94	0.95	[0.27;3.39]	1.05
> 1 surgeries	− 0.71	0.68	1.06	1	.30	0.49	[0.13;1.89]	2.04
PTSD symptoms	− 1.36	0.50	7.53	1	.006	0.26	[0.10;0.68]	3.85
Usual Activities *χ*^2^(3) = 16.06, *p* = .001, Nagelkerke *R*^2^ = 0.11								
Surgery			7.05	2	.03			
1 surgery	− 0.61	0.46	1.73	1	.19	0.54	[0.22;1.35]	1.85
> 1 surgeries	− 1.33	0.50	6.93	1	.009	0.26	[0.10;0.71]	3.85
PTSD symptoms	− 1.08	0.40	7.30	1	.007	0.34	[0.15;0.74]	2.94
Pain/Discomfort *χ*^2^(3) = 18.57, *p* < .001, Nagelkerke *R*^2^ = 0.11								
Surgery			4.63	2	.10			
1 surgery	0.04	0.35	0.01	1	.90	1.04	[0.53;2.05]	0.96
> 1 surgeries	− 0.77	0.41	3.53	1	.06	0.46	[0.21;1.03]	2.17
PTSD symptoms	− 1.35	0.36	13.97	1	< .001	0.26	[0.13;0.53]	3.85
Anxiety/Depression *χ*^2^(3) = 10.70, *p* = .01, Nagelkerke *R*^2^ = 0.12								
Surgery			1.35	2	.51			
1 surgery	0.18	0.46	0.15	1	.70	1.19	[0.49;2.91]	0.84
> 1 surgeries	− 0.42	0.54	0.60	1	.44	0.66	[0.23;1.90]	1.52
PTSD symptoms	− 1.18	0.40	8.63	1	.003	0.31	[0.14;0.68]	3.23
Cognition *χ*^2^(3) = 11.21, *p* = .01, Nagelkerke *R*^2^ = 0.12								
Surgery			3.57	2	.17			
1 surgery	− 0.09	0.46	0.04	1	.85	0.92	[0.37;2.27]	1.09
> 1 surgeries	− 0.88	0.52	2.89	1	.09	0.42	[0.15;1.14]	2.38
PTSD symptoms	− 0.99	0.41	5.74	1	.02	0.37	[0.17;0.83]	2.70

The logistic regression outcome variables are coded as 1 ‘Recovery’ versus 0 ‘Deterioration’. Reference category for Surgery is ‘no surgeries’. Reference category for PTSD symptoms is ‘No substantial PTSD symptoms’

*HRQL* health-related quality of life, *PTSD* post-traumatic stress disorder*, OR* odds ratio

For the Summary Index, *t*-tests showed no significant differences on recalled pre-burn HRQL between the Recovery and Deterioration groups, *t*(255) = − 0.30, *p* = .76, *d* = 0.04. For the VAS, the Deterioration group (*M* = 90.6, SD = 9.1) scored significantly higher than the Recovery group (*M* = 84.2, SD = 12.7) on recalled HRQL, *t*(233.52) = − 4.47, *p* < .001, Cohen’s *d* = 0.59.

## Discussion

This is the first study that describes patterns of change in HRQL after burns using a recalled pre-injury score as the starting point to determine recovery and to investigate whether burn severity and PTSD symptoms increase the likelihood of *not* returning to pre-burn HRQL. Moreover, these findings support the face validity of using an in-hospital recalled pre-injury HRQL as an individual reference point to monitor the patient’s return to pre-burn level of HRQL after a burn injury.

Comparisons between the burn sample and the general population norms showed that although population norms were reached after 18 months, the mean recalled pre-burn levels were not reached, which may reflect individual health loss. This is largely in concert with two pre-burn studies using the SF-36 to measure HRQL [10, 13], but is now also found for the EQ-5D. Other research in burn populations also showed that the norm population’s level is reached after 18 months [9]. However, because the recalled pre-burn level of functioning might not be regained, health loss in burn populations may be underestimated if population norms are used irrespective of pre-burn individualized measures. Our findings regarding the high pre-burn HRQL levels are in line with the broader literature, given that recalled pre-injury HRQL of patients with a variety of injuries produced systematically higher HRQL than population norms both in international research and within the Dutch population [37, 38]. Consequently, these findings also suggest that the use of recalled pre-burn HRQL may further improve the accuracy of recovery estimation models [39].

With reference to the recalled pre-burn baseline scores, four patterns of change in HRQL were defined. Among these patterns, Recovery was most prevalent, followed by the Stable and Deterioration patterns. The Growth pattern occurred only occasionally. For the Summary Index, VAS and most physical domains, the majority of the patients followed a Recovery pattern, whereas in the psychological domains (Anxiety/Depression and Cognition), most patients showed a Stable unaffected pattern. The predominance of the Recovery pattern is mirrored in previous studies using a pre-burn measure that showed a mean decrease in HRQL after the injury, followed by an increase [10, 11, 15]. However, in each domain a subset of patients did not recover to pre-burn baseline levels, especially in the domains Pain/Discomfort, Anxiety/Depression and Cognition, which is in line with previous group level research [9]. Thus, findings extend the literature by showing the existence and extent of other patterns next to Recovery.

Comparing the Recovery and Deterioration pattern, more severely burned patients and patients with PTSD symptoms were less likely to fully recover within 18 months post-injury. These findings are in line with previous research that included a recalled pre-burn measure, that found a positive relationship between larger burn size and physical but not psychological impairment [10] or found a relation between more severe burns and protracted recovery of HRQL in general [11, 13, 15]. These findings support earlier studies at group level [4, 10, 18, 19] by showing that substantial PTSD symptoms were associated with a higher risk for both a long-term affected physical and mental HRQL, whereas more severe burn were associated with a higher risk for a reduced physical HRQL.

Of notice, this study showed that cognitive problems after burns persist beyond 18 months in about 35% of the patients, a health domain that has been scarcely studied in burn patients. A prior study reported cognitive problems in burn patients 2 years post-burn [40] and another study indicated that 16.6% of patients with minor burns and 33.3% of patients with severe burns experienced cognitive problems 5–7 years post-burn [41]. A positive association between PTSD symptoms and cognitive problems may be expected, because adults with PTSD show deficits in cognitive processes such as attention and executive functions [42, 43]. Further research may disentangle possible bio psychological causes of cognitive problems after burns, for example related to the stress response [44] or to sedation effects of mechanical ventilation [45] or anesthesia during surgeries [46].

Regarding the recalled pre-burn measurement, it could be argued that the significantly higher pre-burn HRQL in our sample compared to population norms may reflect an idealization of pre-burn HRQL resulting in an upward bias [e.g., 37]. This phenomenon has been called ‘response shift’ of internal standards, indicating a tendency to inflate the pre-injury assessment by implicit comparison with the poorer health state shortly after the injury [47]. The results regarding pre-burn differences between the Deterioration and Recovery group suggests that the VAS, especially in the Deterioration group with more severely burned patients, may be more prone to an upward bias, whereas the Summary Index (and individual domains) may be more resistant to such an upward bias. However, the possible upward bias in retrospective pre-burn scores does not necessarily mean that the use of population norms is a better reference point to determine recovery, because previous trauma research showed that the upward bias of recall is smaller than the underrepresentation of population norms [48]. Moreover, burn patients in our sample may have had an actual better HRQL than the norm group from the general population, for example, because men and younger persons in the general population have a better HRQL and were somewhat overrepresented in our sample [38, 49]. Also, both the pre- and the 18 months post-burn situation were reported from the plausibly similar shifted post-injury standard of the patient, adding to the validity of the comparisons [50].

The study has some limitations that need to be taken into account. First, the MIDs for the EQ-5D Summary Index and VAS that were used as cut-off point for recovery to pre-injury level stem from other patient populations [32–35]. The strict MIDs partly explain lower frequencies of the Stable pattern on the Summary Index and VAS compared to the individual health domains. Larger MIDs may be more appropriate after burn injury, because of maturation of scars and psychological adjustment occurring during the first years post-burn. Second, we used a self-report questionnaire and not a diagnostic interview to assess PTSD symptoms [51]. The high sensitivity of the IES-R to detect PTSD has been indicated in prior research [30], but specificity of screeners is typically lower [52]. Third, dropout rates were substantial and may bias the found frequency of the different patterns. Also, the group sizes of Deteriorated patients in the three burn severity groups were small in some domains, which may underpower this study for detecting (significant) differences in recovery percentages between the surgery groups in the logistic regression analysis.

The results may encourage clinicians to use a retrospective pre-burn EQ-5D plus Cognition measure as a reference point to monitor individual HRQL recovery over time. Also, the results indicate that patients with more severe burns and patients with elevated PTSD scores may not return to pre-burn level of functioning, and timely interventions for psychological problems may be beneficial for recovery [e.g., 53, 54].

In conclusion, this study supports the face validity of using recalled in-hospital assessed pre-burn HRQL to monitor the patient’s progress in HRQL. Different patterns of change in HRQL were found and patients with more severe burns and substantial PTSD symptoms were less likely to fully recover.

## Electronic supplementary material

Below is the link to the electronic supplementary material.

Supplementary file1 (DOCX 27 kb)

## Data Availability

Data are available upon request from the corresponding author.

## Code availability

Data were analyzed using IBM SPSS Statistics version 24. Syntax is available upon request from the corresponding author.
