# Supplementary material for: Individual recovery of health-related quality of life during 18 months post-burn using a retrospective pre-burn measurement: an exploratory study
Source: Qual Life Res. 2020 Oct 22;30(3):737–49. doi: 10.1007/s11136-020-02678-0 (PMC7952339; doi:10.1007/s11136-020-02678-0)
Supplement: Supplementary file 1 — Supplementary file1 (DOCX 27 kb) [file 11136_2020_2678_MOESM1_ESM.docx]

**Electronic Supplementary Material**

This is supplementary material to the following article:

Individual recovery of health-related quality of life during 18 months post-burn using a retrospective pre-burn measurement: An exploratory study.

E. Boersma-van Dam, R. van de Schoot, H. W. C. Hofland, I. M. Engelhard, N. E. E. Van Loey.

Quality of Life Research

*Correspondence*

Correspondence concerning this content should be addressed to Elise Boersma-van Dam, Association of Dutch Burn Centres, Beverwijk, the Netherlands; E-mail: m.e.boersma-vandam@uu.nl.

This Electronic Supplementary Material consists of two tables containing the logistic regression results for PTSD symptoms measured in hospital and at six months post-burn. The logistic regression results for PTSD symptoms at 3 months post-burn in reported in the main text.

| **Table A** Parameter estimates of the Logistic Regression Analyses for Recovery at 18 months in each domain with PTSD symptoms measured in-hospital | | | | | | | | |
| --- | --- | --- | --- | --- | --- | --- | --- | --- |
|  | *B* | SE | Wald | df | *p* | OR | 95% CI OR | 1/OR |
| Summary Index ꭓ^2^(3) = 27.39, *p* < .001, Nagelkerke R^2^ = 0.13 | | | | | | | | |
| Surgery |  |  | 15.46 | 2 | <.001 |  |  |  |
| 1 surgery | -0.46 | 0.30 | 2.45 | 1 | .12 | 0.63 | [0.35;1.12] | 1.59 |
| >1 surgeries | -1.52 | 0.39 | 15.45 | 1 | <.001 | 0.22 | [0.10;0.47] | 4.55 |
| PTSD in hospital | -1.04 | 0.30 | 12.32 | 1 | <.001 | 0.35 | [0.20;0.63] | 2.86 |
| VAS ꭓ^2^(3) = 6.58, *p* = .09, Nagelkerke R^2^ = 0.04 | | | | | | | | |
| Surgery |  |  | 0.37 | 2 | .83 |  |  |  |
| 1 surgery | 0.11 | 0.29 | 0.14 | 1 | .71 | 1.11 | [0.63;1.98] | 0.90 |
| >1 surgeries | -0.11 | 0.37 | 0.08 | 1 | .77 | 0.90 | [0.44;1.84] | 1.11 |
| PTSD in hospital | -0.74 | 0.30 | 6.29 | 1 | .01 | 0.48 | [0.27;0.85] | 2.08 |
| Mobility ꭓ^2^(3) = 5.77, *p* = .12, Nagelkerke R^2^ = 0.06 | | | | | | | | |
| Surgery |  |  | 2.22 | 2 | .33 |  |  |  |
| 1 surgery | -0.19 | 0.59 | 0.11 | 1 | .75 | 0.83 | [0.26;2.61] | 1.20 |
| >1 surgeries | -0.85 | 0.64 | 1.77 | 1 | .18 | 0.43 | [0.12;1.50] | 2.33 |
| PTSD in hospital | -0.94 | 0.46 | 4.09 | 1 | .04 | 0.39 | [0.16;0.97] | 2.56 |
| Usual Activities ꭓ^2^(3) = 11.02, *p* = .01, Nagelkerke R^2^ = 0.07 | | | | | | | | |
| Surgery |  |  | 8.65 | 2 | .01 |  |  |  |
| 1 surgery | -0.66 | 0.44 | 2.29 | 1 | .13 | 0.52 | [0.22;1.22] | 1.92 |
| >1 surgeries | -1.42 | 0.48 | 8.60 | 1 | .003 | 0.24 | [0.09;0.62] | 4.17 |
| PTSD in hospital | -0.61 | 0.38 | 2.60 | 1 | .11 | 0.54 | [0.26;1.14] | 1.85 |
| Pain/Discomfort ꭓ^2^(3) = 15.94, *p* = .001, Nagelkerke R^2^ = 0.09 | | | | | | | | |
| Surgery |  |  | 6.31 | 2 | .04 |  |  |  |
| 1 surgery | -0.14 | 0.33 | 0.19 | 1 | .67 | 0.87 | [0.45;1.66] | 1.15 |
| >1 surgeries | -0.98 | 0.40 | 5.82 | 1 | .02 | 0.38 | [0.17;0.83] | 2.63 |
| PTSD in hospital | -1.06 | 0.32 | 10.77 | 1 | .001 | 0.35 | [0.18;0.65] | 2.86 |
| Anxiety/Depression ꭓ^2^(3) = 3.87, *p* = .28, Nagelkerke R^2^ = 0.04 | | | | | | | | |
| Surgery |  |  | 2.93 | 2 | .23 |  |  |  |
| 1 surgery | 0.22 | 0.43 | 0.27 | 1 | .60 | 1.25 | [0.54;2.90] | 0.80 |
| >1 surgeries | -0.63 | 0.52 | 1.45 | 1 | .23 | 0.53 | [0.19;1.48] | 1.89 |
| PTSD in hospital | -0.45 | 0.38 | 1.41 | 1 | .24 | 0.64 | [0.30;1.34] | 1.56 |
| Cognition ꭓ^2^(3) = 14.21, *p* = .003, Nagelkerke R^2^ = 0.14 | | | | | | | | |
| Surgery |  |  | 6.40 | 2 | .04 |  |  |  |
| 1 surgery | -0.15 | 0.45 | 0.11 | 1 | .75 | 0.87 | [0.36;2.08] | 1.15 |
| >1 surgeries | -1.20 | 0.51 | 5.49 | 1 | .02 | 0.30 | [0.11;0.82] | 3.33 |
| PTSD in hospital | -1.16 | 0.39 | 8.66 | 1 | .003 | 0.31 | [0.15;0.68] | 3.23 |
| HRQL=Health-related Quality of Life, PTSD = Post-traumatic Stress Disorder, OR = Odds Ratio. The logistic regression outcome variables are coded as 1 ‘Recovery’ versus 0 ‘Deterioration’. Reference category for Surgery is ‘no surgeries’. Reference category for PTSD symptoms is ‘No substantial PTSD symptoms’. | | | | | | | | |

| **Table B** Parameter estimates of the Logistic Regression Analyses for Recovery at 18 months in each domain with PTSD symptoms measured at 6 months | | | | | | | | |
| --- | --- | --- | --- | --- | --- | --- | --- | --- |
|  | *B* | SE | Wald | df | *p* | OR | 95% CI OR | 1/OR |
| Summary Index ꭓ^2^(3) = 42.19, *p* < .001, Nagelkerke R^2^ = 0.21 | | | | | | | | |
| Surgery |  |  | 11.55 | 2 | .003 |  |  |  |
| 1 surgery | -0.42 | 0.32 | 1.78 | 1 | .18 | 0.66 | [0.35;1.22] | 1.52 |
| >1 surgeries | -1.34 | 0.40 | 11.52 | 1 | <.001 | 0.26 | [0.12;0.57] | 3.85 |
| PTSD at 6 months | -1.92 | 0.38 | 25.55 | 1 | <.001 | 0.15 | [0.07;0.31] | 6.67 |
| VAS ꭓ^2^(3) = 12.08, *p* = .007, Nagelkerke R^2^ = 0.07 | | | | | | | | |
| Surgery |  |  | 0.35 | 2 | .84 |  |  |  |
| 1 surgery | 0.12 | 0.30 | 0.15 | 1 | .70 | 1.12 | [0.62;2.02] | 0.89 |
| >1 surgeries | 0.21 | 0.38 | 0.32 | 1 | .57 | 1.24 | [0.59;2.59] | 0.81 |
| PTSD at 6 months | -1.20 | 0.35 | 11.38 | 1 | <.001 | 0.30 | [0.15;0.61] | 3.33 |
| Mobility ꭓ^2^(3) = 7.82, *p* = .05, Nagelkerke R^2^ = 0.09 | | | | | | | | |
| Surgery |  |  | 2.23 | 2 | .33 |  |  |  |
| 1 surgery | 0.37 | 0.62 | 0.36 | 1 | .55 | 1.45 | [0.43;4.91] | 0.69 |
| >1 surgeries | -0.46 | 0.64 | 0.52 | 1 | .47 | 0.63 | [0.18;2.21] | 1.59 |
| PTSD at 6 months | -1.19 | 0.50 | 5.61 | 1 | .02 | 0.30 | [0.11;0.81] | 3.33 |
| Usual Activities ꭓ^2^(3) = 15.97, *p* = .001, Nagelkerke R^2^ = 0.11 | | | | | | | | |
| Surgery |  |  | 7.08 | 2 | .03 |  |  |  |
| 1 surgery | -0.80 | 0.48 | 2.74 | 1 | .10 | 0.45 | [0.17;1.16] | 2.22 |
| >1 surgeries | -1.40 | 0.53 | 7.08 | 1 | .008 | 0.25 | [0.09;0.69] | 4.00 |
| PTSD at 6 months | -1.17 | 0.42 | 7.75 | 1 | .005 | 0.31 | [0.14;0.71] | 3.23 |
| Pain/Discomfort ꭓ^2^(3) = 24.11, *p* < .001, Nagelkerke R^2^ = 0.14 | | | | | | | | |
| Surgery |  |  | 5.51 | 2 | .06 |  |  |  |
| 1 surgery | -0.10 | 0.35 | 0.09 | 1 | .77 | 0.90 | [0.45;1.79] | 1.11 |
| >1 surgeries | -0.92 | 0.41 | 4.89 | 1 | .03 | 0.40 | [0.18;0.90] | 2.50 |
| PTSD at 6 months | -1.60 | 0.38 | 17.91 | 1 | <.001 | 0.20 | [0.10;0.42] | 5.00 |
| Anxiety/Depression ꭓ^2^(3) = 11.19, *p* = .01, Nagelkerke R^2^ = 0.12 | | | | | | | | |
| Surgery |  |  | 1.55 | 2 | .46 |  |  |  |
| 1 surgery | 0.16 | 0.46 | 0.12 | 1 | .73 | 1.17 | [0.48;2.88] | 0.85 |
| >1 surgeries | -0.49 | 0.55 | 0.79 | 1 | .37 | 0.61 | [0.21;1.80] | 1.64 |
| PTSD at 6 months | -1.24 | 0.41 | 9.21 | 1 | .002 | 0.29 | [0.13;0.64] | 3.45 |
| Cognition ꭓ^2^(3) = 17.46, *p* < .001, Nagelkerke R^2^ = 0.17 | | | | | | | | |
| Surgery |  |  | 3.65 | 2 | .16 |  |  |  |
| 1 surgery | -0.04 | 0.47 | 0.01 | 1 | .93 | 0.96 | [0.38;2.42] | 1.04 |
| >1 surgeries | -0.89 | 0.52 | 2.87 | 1 | .09 | 0.41 | [0.15;1.15] | 2.44 |
| PTSD at 6 months | -1.48 | 0.42 | 12.15 | 1 | <.001 | 0.23 | [0.10;0.52] | 4.35 |
| HRQL=Health-related Quality of Life, PTSD = Post-traumatic Stress Disorder, OR = Odds Ratio. The logistic regression outcome variables are coded as 1 ‘Recovery’ versus 0 ‘Deterioration’. Reference category for Surgery is ‘no surgeries’. Reference category for PTSD symptoms is ‘No substantial PTSD symptoms’. | | | | | | | | |
